# AhGLK1 affects chlorophyll biosynthesis and photosynthesis in peanut leaves during recovery from drought

**DOI:** 10.1038/s41598-018-20542-7

**Published:** 2018-02-02

**Authors:** Xing Liu, Limei Li, Meijuan Li, Liangchen Su, Siman Lian, Baihong Zhang, Xiaoyun Li, Kui Ge, Ling Li

**Affiliations:** Guangdong Provincial Key Laboratory of Biotechnology for Plant Development, School of Life Sciences, South China Normal University, Guangzhou, China

## Abstract

Peanut is an important edible oil crop plant whose quality and yield are greatly affected by drought. The process and molecular mechanisms of recovery from drought are also critical to its productivity, but are currently poorly characterized. Here, we investigate the involvement of peanut *AhGLK1* in recovery from drought, and in particular its relationship with *AhPORA*, which encodes a key enzyme in chlorophyll biosynthesis. We found that chlorophyll content, chlorophyll fluorescence, AhPORA protein level and genes related to chlorophyll biosynthesis and photosynthesis declined markedly under drought conditions, but all increased during recovery. Consistent with this, *AhGLK1* expression decreased during water stress and increased when the stress was removed. When *AhGLK1* was transformed into Arabidopsis *glk1glk2* mutant, it increased the survival rate of the mutant during recovery from drought and fully rescued the mutant’s pale-green phenotype. In addition, chlorophyll content and fluorescence, and the expression of genes related to chlorophyll biosynthesis and photosynthesis, were all increased. Bioinformatics analysis and experimental evidence suggested that AhGLK1 augments the expression of *AhPORA* by binding to its promoter. Our findings confirm that AhGLK1 plays a role as a transcription factor that upregulates expression of *AhPORA* during post-drought recovery, thereby stimulating chlorophyll biosynthesis and photosynthesis.

## Introduction

Plants live in extremely variable environments and their survival, growth and productivity are susceptible to various stress factors, such as drought, low temperature, salt, flood, heat, oxidative stress and heavy metal toxicity in both natural and agricultural systems^1^. Among these adverse factors, drought is the most serious environmental factor and is a primary constraint for agriculture worldwide, with the potential to cause significant yield losses and to affect crop quality^2,3^. Many reports have described the response of crops to drought and much effort has been expended on identifying the key genetic and molecular elements of drought resistance in plants^4^. However, stress recovery is also critical to crop survival, growth, yield and quality. The plant’s recovery strategy aims to re-establish the pre-stress metabolic state and, depending on the severity of the stress, to overcome stress-induced senescence mechanisms in the remaining cells and tissues^5^. Despite its importance, however, relatively few studies have been conducted on the underlying mechanisms of plant recovery from drought^6–8^.

Peanut (*Arachis hypogaea*) is one of the most important edible oil crops, and is also an important source of protein^9^. It is widely grown in arid and semi-arid regions of the world, where water stress can significantly affect crop yield and quality^10^. Consequently, much research has been devoted to exploring the molecular details of the drought response in peanut. In our own work, we have characterized the role of some of the transcription factors, such as *AhAREB1*^11^, *AhNAC2*^12^ and *AhNAC3*^13^, involved in this stress response. However, how post-drought growth recovery ability is regulated, and how this ability might be enhanced to improve the quality and yield of peanut, is currently elusive. Nevertheless, a recent study in *Medicago truncatula* suggests that drought recovery processes are regulated differently from those relating to stress tolerance^5^, and therefore investigation of the molecular details of peanut drought recovery are of considerable scientific, and potentially economic, interest.

In a previous study, a yeast two-hybrid screen was conducted using AhHDA1, which participates in the response to drought in peanut, as the bait against a peanut cDNA library, resulting in the identification of 44 AhHDA1-interacting proteins^14^. We were particularly interested in a protein we named AhGLK1 (*Arachis hypogaea* Golden2-like 1), which is implicated in leaf growth recovery from drought. The *AhGLK1* gene (accession number KX168636) is 1212 bp long and contains six exons, which encode a 404-residue protein with a conserved MYB domain. AhGLK1 is located in the nucleus and is a transcription activation factor with a high degree of sequence relatedness to Golden2-like (GLK) transcription factors (TFs) from other plant species^14^.

GLK TFs are members of the GARP family of MYB TFs and are potent positive regulators of photosynthesis-related genes in numerous plants^15,16^. They regulate chloroplast development and maintenance in Arabidopsis, maize, and the moss *Physcomitrella patens* by binding directly to the promoter sequences of a number of genes that are required for chloroplast development and light-harvesting functions^16–18^. There are two *GLK* genes in Arabidopsis, designated *GLK1* and *GLK2*, which function redundantly to regulate chloroplast biogenesis. An early study revealed that the size of chloroplasts and the numbers of thylakoid lamellae in a *glk2* mutant were both smaller than in WT^19^. As expected in a strain with poorly developed chloroplasts, the *glk1glk2* double mutant exhibits a pale-green phenotype^18^. Deficiency of both *GLK1* and *GLK2* leads to a marked decrease in chlorophyll biosynthesis genes in leaves^16,18^. When a GFP-GLK chimeric construct was transformed into an Arabidopsis *glk1glk2* double mutant, it complemented the mutant’s pale-green phenotype and restored transcription levels of the *Lhcb* genes, which encode the proteins of the LHCII light-harvesting complex intrinsic to photosystem II (PSII)^20^. Furthermore, overexpression of GLK proteins induces strong expression of chlorophyll-related genes, with ectopic chlorophyll accumulation in non-photosynthetic organs such as rice calli, root cells and fruits^21–23^.

The light energy absorbed by chlorophyll molecules and other pigments drives the photochemical reactions of photosynthesis. This energy is used for photosynthetic electron transport, but can also be lost through thermal dissipation or be re-emitted as chlorophyll fluorescence^24^. Chlorophyll fluorescence is an indicator of photosynthetic efficiency, and can be determined by measurement of various parameters of PSII photochemistry, such as Fv/Fm, the ratio of variable to maximum fluorescence after dark adaptation, which shows the maximum quantum yield of PSII and is used to monitor stress in plants^25^. This is a very sensitive indicator of photosynthetic performance and has proven to be a powerful tool for the accurate diagnosis of the abiotic and biotic stresses that plants are exposed to, e.g. latent manganese deficiency and ultraviolet B radiation, among others^26–28^. Much *in vivo* research has shown that drought stress leads to considerable damage to the oxygen evolving center of PSII, leading to the inactivation of the PSII reaction center and altered chlorophyll fluorescence^29^.

Chlorophyll biosynthesis is controlled by three structurally related NADPH: protochlorophyllide oxidoreductases (PORs) in *A. thaliana* called *PORA*, *PORB* and *PORC*^30–32^. Each POR catalyzes the light-dependent reduction of protochlorophyllide *a* to chlorophyllide *a*, which is subsequently converted to chlorophyll during photomorphogenesis. Recently, it was claimed that PORA is not only transiently involved in initiating chlorophyll biosynthesis during illumination of etiolated seedlings, but is also essential for normal growth and plant development^33^. *AhPORA*, which is designated *Aradu.10012670* in PeanutBase (www.peanutbase.org), is one of the *POR* genes involved in chlorophyll biosynthesis in peanut.

Here, because AhGLK1 is highly related to other GLK proteins, we hypothesized that it may bind to promoter sequences of a number of genes that are involved in chlorophyll biosynthesis and light-harvesting functions, thereby stimulating the chlorophyll biosynthesis and photosynthesis genes required for peanut recovery from drought. To test this hypothesis, we first determined the relative water, chlorophyll content, chlorophyll fluorescence, expression of *AhGLK1* (and its cognate protein), the expression of genes related to chlorophyll biosynthesis and photosynthesis, and AhPORA protein expression level in peanut plants under water-limiting conditions and during the recovery process. Next, *AhGLK1* was transformed into Arabidopsis *glk1glk2* mutants to demonstrate its effect on chlorophyll biosynthesis and photosynthesis, and on survival rate and growth during recovery from drought. It was observed that expression of *PORA* and its cognate protein in the *AhGLK1/glk1glk2* strain are highly upregulated, suggesting that *AhPORA* is one of the target genes of AhGLK1. In addition, we carried out bioinformatics on the *AhPORA* promoter to examine whether AhGLK1 might regulate the *AhPORA* gene. Our study will contribute to an improved understanding of peanut growth recovery from drought, which is potentially important for improving crop yield and quality.

## Results

### AhGLK1 is implicated in peanut growth recovery from drought

**Figure 1:**
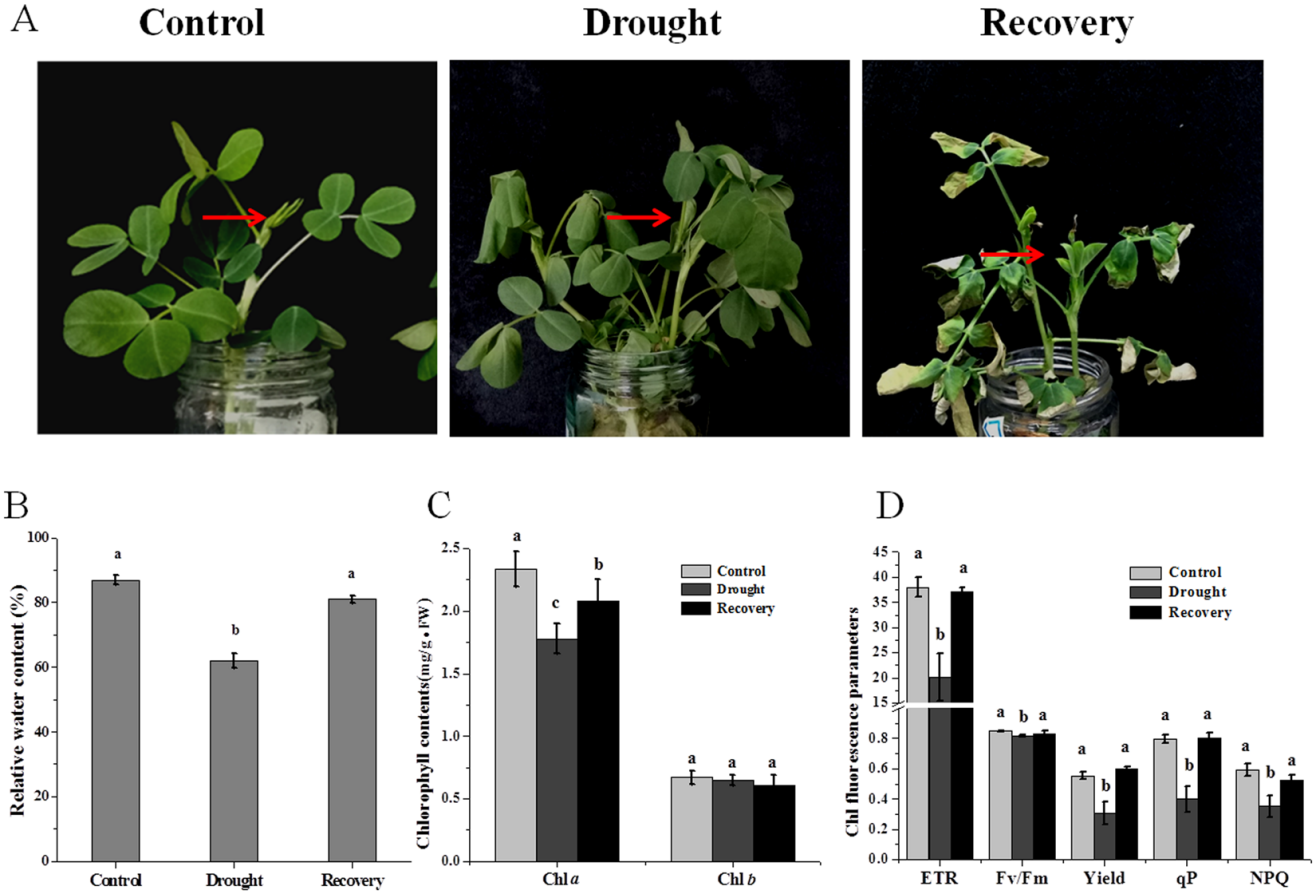
Morphological and physiological changes in peanut plant during drought stress and recovery. (**A**) The morphological characteristics of control, drought and recovery peanut plants at the four-leaf-stage. Arrows indicate the leaf type chosen for experiments. (**B**) The relative water content of control, drought and recovery peanut leaves. (**C**) The chlorophyll content of control, drought and recovery peanut leaves. (**D**) The chlorophyll fluorescence parameters of control, drought and recovery peanut leaves. An average of three biological replicates is shown. Error bars represent SD. Different letters (a,b,c) represent a significant difference within groups.

During drought stress, the morphological and physiological responses of plant leaves are critical in reducing water loss and promoting efficient use of water. When peanut plants were subjected to 10% PEG for 5 h, their leaves began to wilt, and were wilting severely after 24 h (Fig. 1A). However, after removal of the stress and recovery for 7 d, new leaves had appeared and wilting leaves had regained their turgor. Measurements of the relative water content were consistent with these observations (Fig. 1B). It has been reported that chlorophyll content notably declines under drought stress, and that the decrease in total chlorophyll content is mainly due to the decrease in Chl *a*^34–36^. Accordingly, we found that Chl *a* content was dramatically reduced in peanut during water stress, and that levels were partially restored during recovery. In contrast, Chl *b* content was unchanged throughout (Fig. 1C).

**Figure 2:**
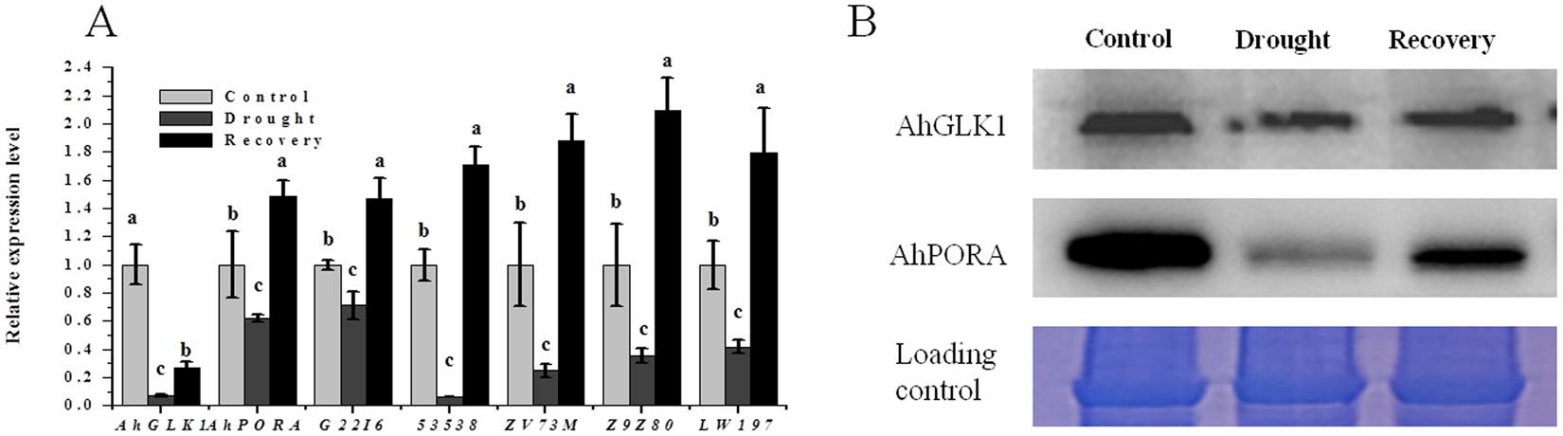
The expression of *AhGLK1* and genes related to chlorophyll biosynthesis and photosynthesis, AhGLK1 and AhPORA protein level during drought stress and recovery growth. (**A**) Relative expression of *AhGLK1*, *AhPORA*, *Aradu.G22I6* (encoding ribulose bisphosphate carboxylase), *Aradu.53538* (encoding light-harvesting chlorophyll B-binding protein 3), *Aradu.ZV73M* (encoding the CHLH subunit of magnesium chelatase), *Aradu.Z9Z80* (encoding a glutamyl-tRNA reductase family protein), and *Aradu.LW197* (encoding chlorophyllide A oxygenase) determined by qRT-PCR. (**B**) AhGLK1 and AhPORA protein expression levels were determined by immunoblotting peanut plant extracts with anti-AhGLK1 and anti-PORA antibody. The average of three biological replicates is shown. Error bars represent SD. Different letters (a,b,c) represent a significant difference within groups.

In response to drought, the chlorophyll fluorescence parameters electron transport rate (ETR), Fv/Fm value, photochemical quenching (qP), non-photochemical quenching coefficient (NPQ) and photochemical yield of PSII in the light (yield) were reduced. ETR, qP, NPQ and yield decreased dramatically by about 47.0%, 44.9%, 49.8% and 40.4%, respectively, compared to the control, although Fv/Fm changed only slightly. These results likely indicate that the activity of PSII was reduced when the stress was imposed. When growth had resumed, the chlorophyll fluorescence parameters, and by inference PSII activity, were largely restored to pre-stress levels (Fig. 1D). Accordingly, we tested the expression of *AhGLK1, AhPORA*, *Aradu.G22I6* (encoding ribulose bisphosphate carboxylase), *Aradu.53538* (encoding light-harvesting chlorophyll B-binding protein 3), *Aradu.ZV73M* (encoding the CHLH subunit of magnesium chelatase), *Aradu.Z9Z80* (encoding a glutamyl-tRNA reductase family protein), and *Aradu.LW197* (encoding chlorophyllide A oxygenase). The transcription of all genes was notably reduced under drought conditions, but increased after recovery; AhGLK1 protein levels reflected the expression of its cognate gene. In agreement with the *AhPORA* gene expression profile, the AhPORA protein level was negatively affected by drought, but was increased after recovery (Fig. 2A and B). These results prompted us to hypothesize that *AhGLK1* plays a role in the resumption of growth after drought and to investigate the effect of *AhGLK1* on chlorophyll biosynthesis and photosynthesis.

### *AhGLK1* is involved in recovery growth and affects chlorophyll biosynthesis and photosynthesis in Arabidopsis

**Figure 3:**
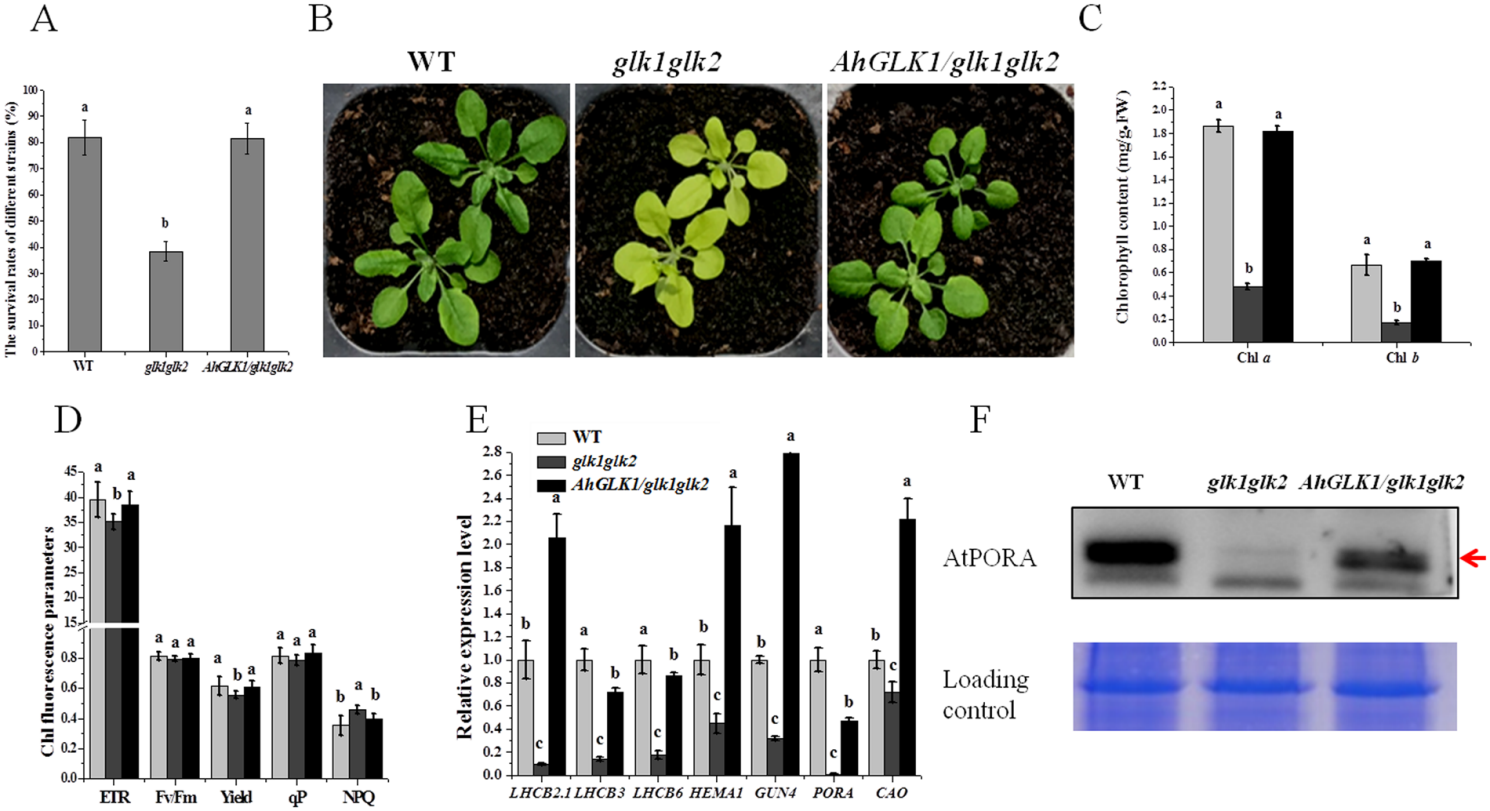
*AhGLK1* enhances the survival rate of Arabidopsis *glk1glk2* mutants in recovery from drought and affects chlorophyll biosynthesis and photosynthesis in *Arabidopsis*. (**A**) The survival rates of WT, *glk1glk2* and *AhGLK1/glk1glk2 Arabidopsis* plants during recovery from 10 days of drought stress. (**B**) The morphology of two-week-old WT, *glk1glk2* and *AhGLK1/glk1glk2 Arabidopsis* plants. (**C**) The chlorophyll content of WT, *glk1glk2* and *AhGLK1/glk1glk2* plants. (**D**) The chlorophyll fluorescence parameters of WT, *glk1glk2* and *AhGLK1/glk1glk2* plants. (**E**) Relative expression of genes related to chlorophyll biosynthesis and photosynthesis analyzed by qRT-PCR. (**F**) AtPORA protein expression level was determined by immunoblotting Arabidopsis plant extracts with anti-PORA antibody. The average of three biological replicates is shown. Error bars represent SD. Different letters (a,b,c) represent a significant difference within groups. Red arrow represents AtPORA bands.

We transformed *AhGLK1* into the Arabidopsis *glk1glk2* double mutant and determined the survival rates of the *glk1glk2* double mutant, *AhGLK1/glk1glk2* and WT strains during recovery from 10 days of drought. As anticipated, *AhGLK1/glk1glk2* showed about a two-fold higher survival rate than *glk1glk2* plants, which was comparable to that of WT (Fig. 3A). The result implies that *AhGLK1* can improve drought resistance and plays an important role in the recovery growth process. We next investigated the possible mechanism of *AhGLK1* function in this context.

The predicted structure of the AhGLK1 protein appears to be well-conserved, as judged by comparison of AtGLK1 and AtGLK2 ribbon diagrams (Fig. S1). To identify whether AhGLK1 functions similarly to Arabidopsis GLK TFs in the context of chlorophyll biosynthesis and photosynthesis, we transformed *AhGLK1* into a *glk1glk2* double mutant. Two weeks later, as anticipated, the control *glk1glk2* plants exhibited pale-green leaves of a reduced size and remarkably lower Chl *a* and *b* content than WT. By contrast, *AhGLK1/glk1glk2* plants had green leaves and a distinctly higher Chl *a* and *b* content, essentially the same as WT plants (Fig. 3B and C). These results suggest that *AhGLK1* is sufficient to fully rescue the *glk1glk2* mutant phenotype and thereby restore Chl *a* and *b* contents.

We next asked whether *AhGLK1* influences chlorophyll fluorescence parameters and found that, in *AhGLK1/glk1glk2* plants, Fv/Fm values, ETR, yield, qP and NPQ were very similar to WT, whereas in *glk1glk2* plants ETR and yield were significantly lower than in WT, and NPQ was higher (Fig. 3D). These results show that *AhGLK1* can influence photosynthetic efficiency and led us to hypothesize that *AhGLK1* can regulate genes related to photosynthesis and chlorophyll biosynthesis, in a similar way to the GLK TFs. Consequently, we tested the expression of the *LHCB2.1, LHCB3, LHCB6* genes, which encode members of LHCII, as well as four genes regulating key enzymatic steps in the chlorophyll biosynthesis pathway: *HEMA1* (glutamyl-tRNA reductase, which catalyzes the rate-limiting and first commitment step in tetrapyrrole biosynthesis), *GUN4* (required for efficient Mg-chelatase activity), *CAO* (which catalyzes the conversion of chlorophyllide *a* to chlorophyllide *b*) and *PORA*. The *glk1glk2* mutants exhibited reduced transcription levels for all seven genes, as reported previously, while in *AhGLK1/glk1glk2* plants expression was about three- to forty-fold higher than in the double mutants. Among these genes, *PORA* was extremely highly upregulated (approximately forty-fold) compared to *glk1glk2* plants. Consistent with the gene expression, PORA protein expression level was significantly increased in *AhGLK1/glk1glk2* plants, implying it is one of the target genes of *AhGLK1* (Fig. 3E and F). It was somewhat surprising, however, that not all genes responded equivalently to *AhGLK1* overexpression and that their expression differed from WT: while expression levels of *LHCB2.1, GUN4, HEMA1*, and *CAO* were approximately two- to three-fold higher than in WT plants, *LHCB3, LHCB6* and *PORA* showed lower expression levels than WT.

### AhGLK1 probably regulates the expression of *AhPORA* by binding to its promoter

**Figure 4:**
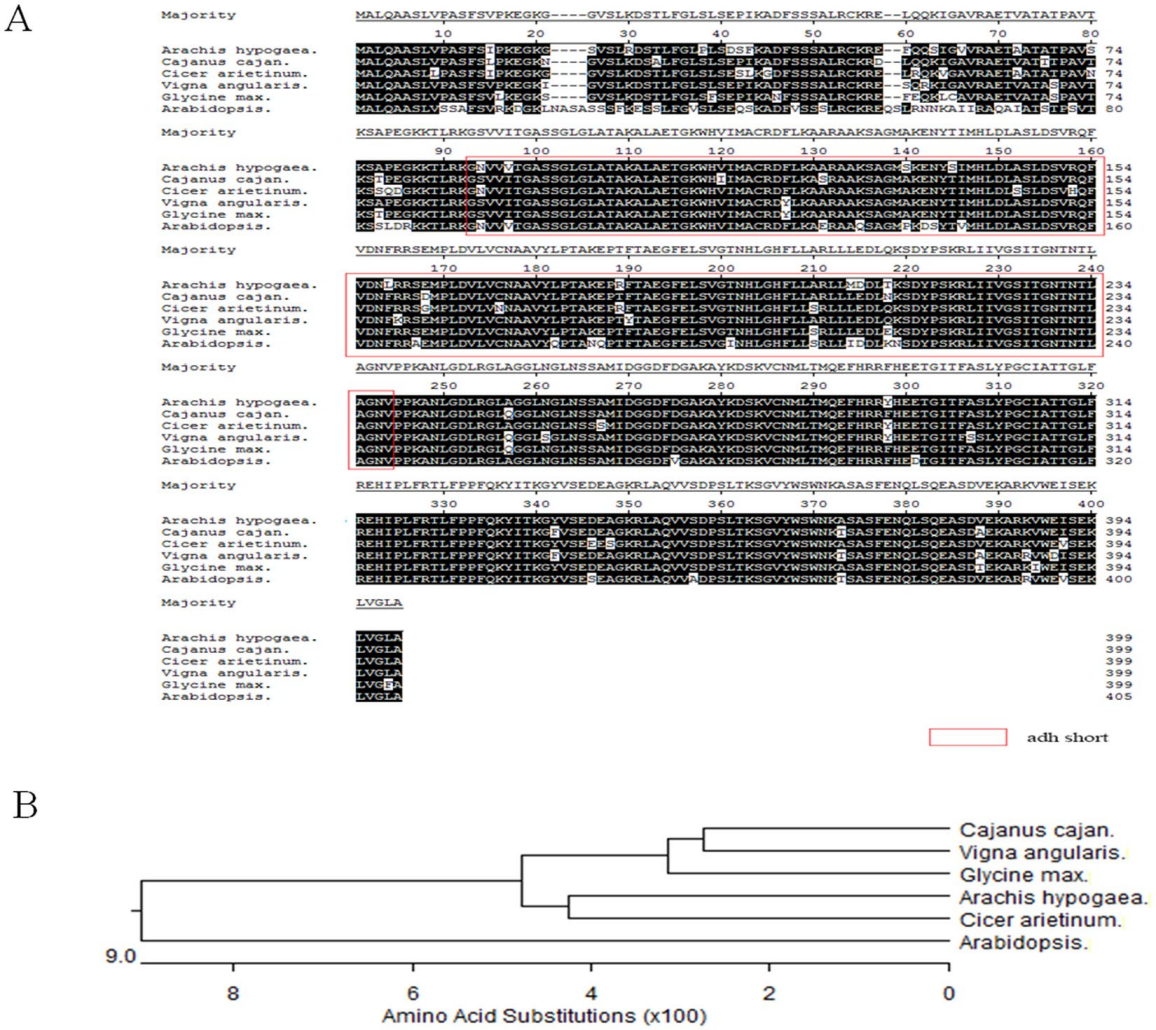
Analysis of PORA amino acid sequence. (**A**) AhPORA amino acid sequence was aligned with other PORA from various plants by MegAlign software. Shaded (solid black) amino acid residues match the consensus. The conserved adh_short domain is shown in a red box. GenBank accession number: Cajanus cajan (XP_020228192.1), Cicer arietinum (XP_004487684.1), Vigna angularis (XP_017425176.1), Glycine max (XP_003540452.1), Arabidopsis (NC_003076.8). (**B**) Phylogenetic tree analysis of amino acid sequences of AhPORA and other plant PORAs.

**Figure 5:**
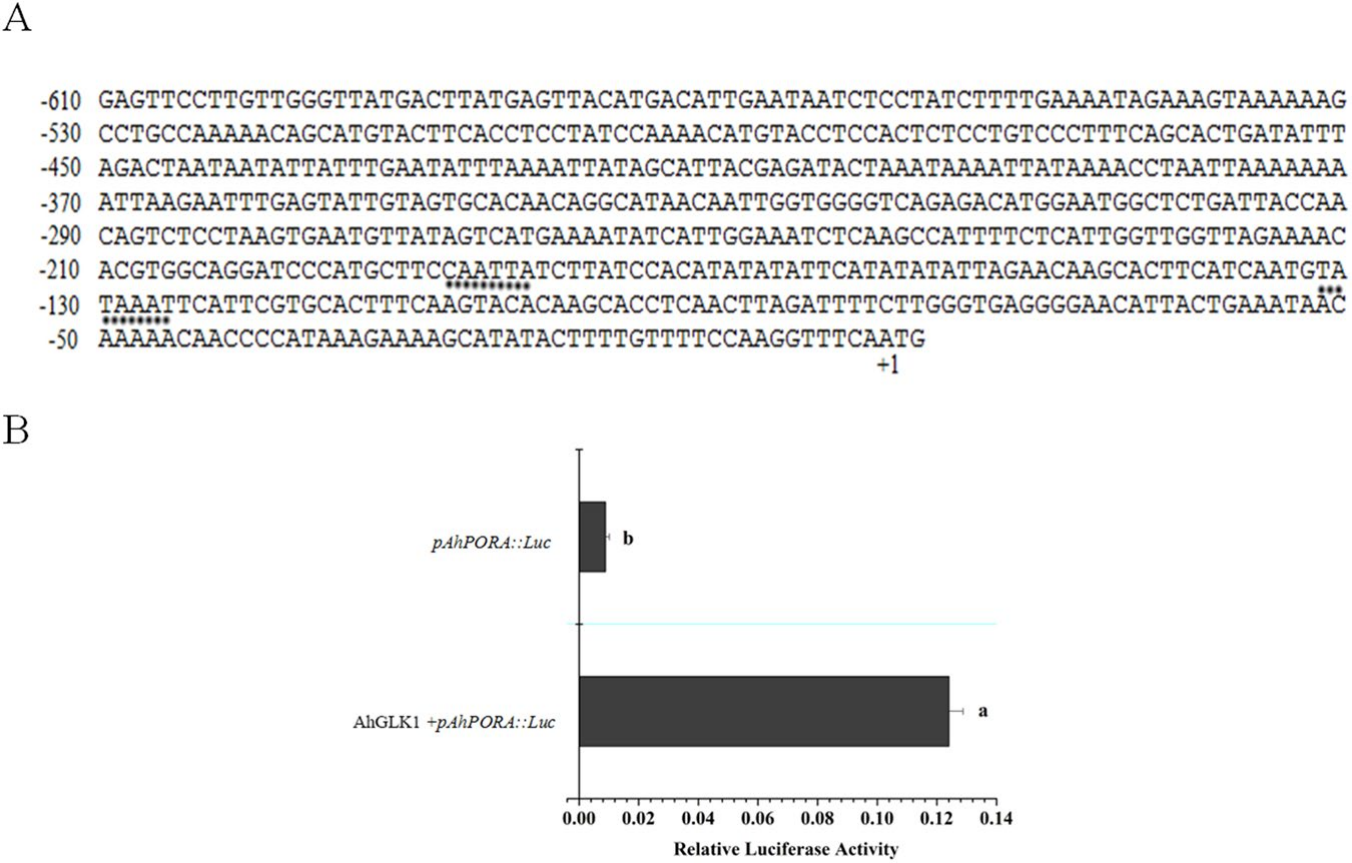
AhGLK1 enhances *AhPORA* promoter activity. (**A**) Nucleotide sequence of the *AhPORA* promoter. The A nucleotide of the transcriptional start site is assigned position +1 in the nucleotide sequence, and the nucleotides upstream of position +1 are presented as negative numbers. The basic element TATA and CAAT boxes are indicated by dotted underlines. (**B**) The luciferase assay was performed by co-transforming reporter plasmid *pAhPORA:Luc* and effector plasmid *pAhGLK1* into *Arabidopsis* mesophyll protoplasts. Relative LUC activity was tested and compared with the control without effector by one-way ANOVA. Bars indicate the standard errors of three replicates. Different letters (a,b) represent a significant difference within groups.

As shown above, *AhGLK1* affects chlorophyll biosynthesis and markedly enhances the expression of *PORA* and its cognate protein, a key enzyme in chlorophyll biosynthesis in Arabidopsis. Thus, we reasoned that *AhPORA* is one of the target genes of AhGLK1. The putative AhPORA protein comprises 399 amino acids and exhibits 82.96%, 75.00%, and 72.68% identity with Arabidopsis PORA, PORB, and PORC, respectively (Fig. S2). The markedly greater similarity to Arabidopsis PORA substantiates our designation of the peanut enzyme as the A isotype (i.e. AhPORA). AhPORA also shows a high degree of similarity to PORAs from other plant species, and contains the conserved adh_short domain, which characterizes the short-chain dehydrogenase/reductase family (Fig. 4A). AhPORA is most similar to counterparts in eudicots, especially *Cicer arietinum* (Fig. 4B). The *AhPORA* promoter sequence was cloned as a 610 bp PCR product from a genomic DNA library and then sequenced. As shown in Fig. 5A, two putative basic *cis*-acting elements were found in the *AhPORA* promoter, a TATA box (−132, −126) and a CAAT box (−188, −183). The TATA box is required to ensure precise initiation of transcription. The CAAT box, a key element in regulating the frequency of transcription, can be found in the promoters of many eukaryotic genes. In addition, the *AhPORA* promoter contains an ABRE motif (−210, −204, ACGTGGC) involved in abscisic acid responsiveness. In addition to the ABRE element, a G box (−214, −204, ACACGTGGC), involved in light responsiveness, and an MRE motif (−386, −380, AACCTAA), which functions as a MYB-recognizing element, were also found. MREs regulate multiple processes, including embryogenesis, flower morphology, light responsiveness and mechanical damage.

To assess whether AhGLK1 regulates the expression of *AhPORA*, we co-transformed *AhGLK1* and *pAhPORA-Luc* vectors into WT Arabidopsis mesophyll protoplasts. We found that AhGLK1 significantly upregulates the expression of *pAhPORA-Luc*: *AhPORA* promoter activity increased about 13-fold compared to the control (Fig. 5B), consistent with our hypothesis.

**Figure 6:**
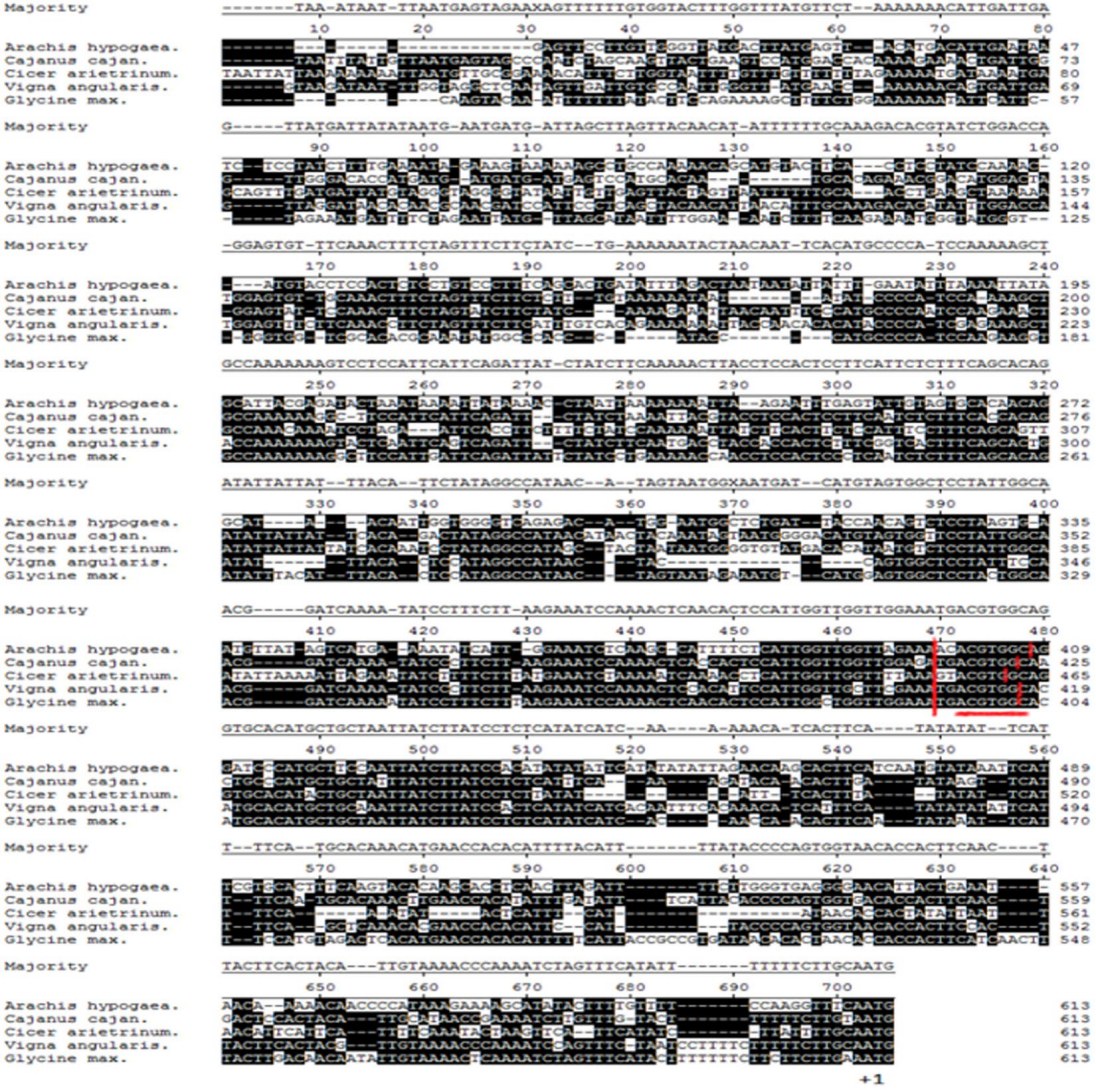
Analysis of *PORA* promoter sequences of various plant species. *AhPORA* promoter sequence was aligned with other PORA from various plants by MegAlign software. Nucleotides matching the consensus are shaded (with solid black). The ABRE (red underline) and G box *cis*-acting elements (between two vertical red lines) were predicted by PlantCARE.

To further investigate the features of the *PORA* promoter, a comparative analysis was performed on the promoter sequences of *A. hypogaea*, *Cajanus cajan*, *Cicer arietinum*, *Vigna angularis* and *Glycine max*. The *PORA* gene sequences of these species are 85–88% identical, and therefore we were able to align the respective promoters and search for *cis*-acting regulatory elements using PlantCARE, a database of plant promoters. The promoter sequences share conserved ABRE (underlined in red) and G-box *cis*-acting elements (Fig. 6), suggesting that *PORA* expression is at least partially regulated by ABA. Some promoters also have *cis*-regulatory elements that implicate other plant hormones, such as ethylene, auxin, MeJA and salicylic acid, in the regulation of their respective genes. Notably, all promoters contain several regulatory elements involved in the response to light (Table S1), which is consistent with the role of *PORA* in chlorophyll biosynthesis. The *C. cajan* promoter, for example, has as many as six light-responsive elements. However, which *cis*-acting elements are responsible for the regulation of *PORA* expression remains to be demonstrated.

## Discussion

It is well documented that drought is one of the environmental factors most damaging to crop yields. Drought stress leads to detrimental changes in all plant organs at the morphological, physiological, biochemical and metabolic levels^37^, with photosynthesis and cell growth primarily affected^38^. As recovery from drought is also important for plant productivity, some recent reports have investigated drought recovery in several species. A study of physiological, metabolic and proteomic data in the model legume *M. truncatula*^5^ revealed that recovery differs considerably from, and is not simply the reverse of, drought acclimation. Although most drought-responsive proteins reverted to control levels directly after rewatering, there were also changes in a broad new set of root and shoot proteins, suggesting that regulatory mechanisms for drought and recovery are independent. These authors therefore provide evidence for a novel, thus far undetected, metabolic remobilization network that is involved in recovery rather than adjustment to stress. In another study, Iovieno *et al*. identified the transcriptomic changes that control physiological adjustments during drought stress and recovery in tomato plants^39^, while Bisaga *et al*. presented the first large scale molecular analysis of the white clover response to drought stress and rehydration^40^. However, to date, there are no reports on the recovery of peanut from drought.

During the recovery of plants from drought, there are conspicuous changes in the leaves, including the appearance of new leaves, unfolding of slightly damaged leaves and shedding of severely damaged leaves^41,42^. Therefore, we concentrated our attention on the peanut leaves, including morphological and physiological effects, during drought stress and the subsequent recovery process. It was observed that mature peanut leaves were unable to completely recover from drought (exposed to 10% PEG for 24 h): during recovery, the edges of this type of leaf became yellow and dry. However, the intact leaves indicated by red arrows in Fig. 1A were able to revive fully after drought stress and, accordingly, we chose this type of leaf for our experiments. These observations imply that different kinds of leaf have variable capacities for recovery; this interesting phenomenon will be the subject of future studies.

Photosynthetic performance is extraordinarily sensitive to environmental stress and is therefore an important indicator of drought^43^. The early leaf senescence in plants subjected to water stress accelerates the degradation of photosynthetic pigments due to the deterioration of thylakoid membranes, and consequently photosynthesis rates drop drastically^4,10^. Furthermore, drought stress is known to alter the Chl *a* fluorescence kinetics and hence impairs the PSII reaction center^44^. Indeed, drought adversely affects the functionality of both PSII and PSI, resulting in decreased electron transport through both systems^40,45^. Moreover, under both short- and long-term drought conditions, chlorophyll content, chlorophyll fluorescence and relative water content gradually decrease in *A. thaliana*^46^. Our results confirm that, during drought, relative water content, chlorophyll content and chlorophyll fluorescence also decrease in peanut (Fig. 1B–D). Stress factors are believed to reduce levels of photosynthetic pigments, so that both PSI and PSII absorb light less efficiently and hence exhibit reduced photosynthetic capacity^47^. Accordingly, ETR, qP and yield are significantly reduced under drought conditions, indicating that a large fraction of the absorbed irradiance is not used photochemically^29^. Desiccation can affect both the donor side and acceptor side of PSII^29^. Fv/Fm is not dramatically reduced, indicating OEC is not damaged in this drought condition. Consequently, enhanced reduction of the plastoquinone pool on the PSII acceptor side, which can be attributed to retarded electron transport from PSII to downstream components such as cytochrome b6f and PSI leads to a reduced qP. Under drought stress, the change of NPQ is varied in different kinds of plant, e.g. drought stress has no significant effect on the NPQ in Maize seedlings^48^. However, NPQ is markedly increased in Arabidopsis under both short- and long-term drought conditions^46^. In this study, NPQ is reduced, suggesting that the intact leaves of peanut may protect them from photodamage via other protective mechanism, not by transforming excessive light energy into heat. After 7 d recovery from drought, peanut plants show new leaf growth, and the relative water content, chlorophyll content and chlorophyll fluorescence parameters all improve dramatically, demonstrating that chlorophyll biosynthesis and PSII activity have recovered. Chlorophyll fluorescence parameters are restored almost to pre-stress levels, indicating the electron transport is not inhibited and that photosynthetic rate and efficiency are enhanced during the recovery process. We can therefore deduce that the structure and functions of PSII are not severely impaired under our drought conditions, despite the dramatic decline in expression of relevant genes. Accordingly, the expression of *AhGLK1* and *AhPORA* and their cognate proteins, together with other genes related to photosynthesis and chlorophyll biosynthesis, are also increased notably. These results signify that *AhGLK1* might be involved in recovery of growth after water stress and that *AhPORA*, which is critical for chlorophyll biosynthesis, may be an important target of AhGLK1.

As shown in Fig. 3A, we demonstrated here that overexpression of *AhGLK1* in the Arabidopsis *glk1glk2* double mutant can enhance its drought tolerance which contributes to recovery growth, implying that *AhGLK1* plays an important role in recovery growth. The *glk1glk2* plants usually have pale-green leaves and depressed chlorophyll levels, but transformation with *AhGLK1* entirely restored the colour of the plants, as well as the Chl *a* and *b* content, to those of WT plants. It has been suggested that GLK function may optimize photosynthetic capacity by integrating responses to variable environmental and developmental conditions^16^. We found that chlorophyll fluorescence parameters almost fully recovered in *AhGLK1/glk1glk2* plants and that transcript levels of genes associated with chlorophyll biosynthesis and light-harvesting were dramatically enhanced. We also observed that, although Fv/Fm values and qP are not significantly different in *glk1glk2* plants compared to WT, ETR and fluorescence yield are markedly lower in the mutants. As ETR and yield reflect photosynthetic rate, this shows that photosynthesis is weaker in *glk1glk2* plants. Consequently, *glk1glk2* plants transform excessive chlorophyll fluorescence into heat to protect them from photodamage, leading to higher NPQ than in WT. The observed reduction in chlorophyll content and PSII efficiency is consistent with a general downregulation of genes involved in chlorophyll biosynthesis and light harvesting in the double mutants. These results confirm that AhGLK1 has a similar function to GLKs in other species, i.e. it regulates genes required for chlorophyll biosynthesis and light-harvesting functions.

We also demonstrated here that *AhPORA* can be regulated by AhGLK1, likely by binding to its promoter. Bioinformatics analysis showed that the *AhPORA* promoter contains an MRE motif, a MYB-recognizing element, and it is probably not a coincidence that AhGLK1 contains a conserved MYB domain. Under drought stress, *AhPORA* expression and cognate protein level markedly decrease, correlating with reduced expression of *AhGLK1* and its cognate protein. A similar correlation was observed during recovery from drought, when expression of both genes and their proteins increased. Therefore, we tentatively propose that AhGLK1 can recognize and bind to the MRE motif in the *AhPORA* promoter and thereby activate *AhPORA*. Other TFs are known to be involved directly or indirectly in the regulation of genes involved in photosynthesis; e.g. LONG HYPOCOTYL 5 (HY5), a bZIP-type, is mainly involved in the regulation of *CAB* gene expression by light, although it may also exhibit a significant role in abiotic stress tolerance^49^. Taken together, our results show that, like other GLK TFs, AhGLK1 is a TF with a positive effect on growth during recovery from drought and that it regulates genes that promote the biosynthesis of chlorophyll and stimulate PSII activity.

In conclusion, *AhGLK1* plays an important role in the recovery of chlorophyll biosynthesis and photosynthesis in peanut following drought stress. AhGLK1 probably binds to the promoter of *AhPORA* and upregulates its expression, leading to increased chlorophyll content. However, whether AhGLK1 regulates other photosynthesis-related genes by binding to their promoters, and other details of the mechanisms by which *AhGLK1* affects peanut recovery from drought, remain to be determined. We showed previously that AhGLK1 interacts with AhHDA1 that has been approved to respond to drought, so we plan to explore the significance of this interaction in more detail in the context of post-drought recovery and the regulation of *AhPORA* in peanut.

## Materials and Methods

### Plant materials and growth conditions

Peanut seeds were sown and grown as described^50^. The Yueyou 7 line was provided by the Crop Research Institute, Guangdong Academy of Agricultural Sciences, China. PEG 6000 (w/v) was used to simulate the effect of drought stress. Four-leaf-stage (10–12 days after sowing) plants were carefully removed from the soil mixture and then grown in 10% PEG for 24 h to dehydrate them. They were then transferred to water to recover for 7 d. Peanut leaf samples (100 mg) subjected to drought stress and recovery were rapidly Day 0 of the experiment was taken as the control. Arabidopsis *glk1glk2* double mutant seeds were obtained from the Arabidopsis Biological Resource Center (ABRC; http://www.arabidopsis.org). The *AhGLK1/glk1glk2* seeds were produced as described below (see Plasmid construction and Arabidopsis transformation). All seeds were surface sterilized in 10% sodium hypochlorite for 7 min, and twice by 75% ethanol, for no more than 1 min each time, followed by washing five times in ddH_2_O. Floating seeds were discarded. The remaining seeds were suspended in ddH_2_O and sown on 0.5× Murashige and Skoog (MS) medium with 0.8% agar containing 2% sucrose. Excess water was allowed to evaporate. After 2 d stratification at 4 °C, the germinated seeds were grown in a climate chamber under a daily cycle of 16 h light and 8 h dark at 20 ± 2 °C. Three days later, the seedlings were transferred to soil and allowed to grow on.

### Plasmid construction and Arabidopsis transformation

The cDNA encoding *AhGLK1* was amplified from peanut and cloned into the modified *pCanG* plasmids (*p35S:eGFP*) to obtain *p35S:AhGLK1-eGFP* by a homologous recombination method. The primers used to construct this plasmid are as follows: F (5′–3′): GGGTTCGAAATCGATGGATCCATGCTTGCGGTGTCACCTTTG; R (5′–3′): GTCCTAGGCTACGTAGGATCCTTAATTAAGCACAGGAGTTGC. The floral dip method was used to transform the plasmids into the *glk1glk2* Arabidopsis strain as previously reported^51^. Dark-green F1 progeny were selected on 0.5× MS medium containing 50 mg/L kanamycin B (Sigma-Aldrich) and selfed. The F2 generation was screened for dark-green individuals representing *AhGLK1/glk1glk2* homozygotes. These individuals were selfed, and F3 seeds were then sown on kanamycin B to identify lines homozygous for *35 S::AhGLK1/glk1glk2*.

### Measurement of survival rate of Arabidopsis during recovery from drought

Drought stress was imposed on three-week-old Arabidopsis plants by withholding water for 10 days according to the method of Sperdouli and Moustakas^52^, and this was followed by re-watering for 4 days. Surviving plants of each strain, i.e. the *glk1glk2* double mutant, *AhGLK1/glk1glk2* and WT strains, were counted and the survival rate was calculated.

### Measurement of relative water and chlorophyll content in leaf

Fresh leaves (100 mg) were harvested from control, drought stress and recovery peanut plants or four-week-old transgenic Arabidopsis. Whole leaves were pulverized, then 10 mL 80% (v/v) acetone was added to extract chlorophyll in the dark for 48 h. Debris was removed by centrifugation at 10,000 × g for 5 min. The absorbance of the supernatant at 663 and 645 nm was measured using an ultraviolet spectrophotometer (UV-2550, Shimadzu, Japan). The chlorophyll (*a* and *b*) concentration of the samples were determined as described^53^. Relative water content of peanut was measured as described^54^. Turgid weight was measured after maintaining leaves in distilled water in the dark at 4 °C overnight, until leaves reached a constant weight. Dry weight was determined after incubation of the turgid leaf at 70 °C for 24 h.

### Chlorophyll fluorescence measurements

Four-leaf-stage peanut plants from control, drought stress and recovery experiments, and two-week-old Arabidopsis seedlings, were dark-adapted for at least 30 min before measurement. Chlorophyll fluorescence was measured using a hand-held portable fluorescence detector (PAM-2100, Germany). The minimal fluorescence level (F_0_) and the maximal fluorescence level (Fm) were measured in dark-adapted leaves using a 6000 μmol m^−2^ s^−1^ saturating pulse. The actinic light intensity used for the PAM analysis was 200 μmol m^−2^ s^−1^. After photoactivation, Fv/Fm value, qP, ETR, NPQ and yield were determined.

### Quantitative real-time PCR (qRT-PCR)

Total RNA was extracted as described by Li^55^. Reverse transcription was carried out using a Prime Script TM RT Reagent Kit with gDNA Eraser (Perfect Real Time, TaKaRa). SYBR qPCR Master Mix (Perfect Real Time, TaKaRa) was used according to the manufacturer’s instructions with an ABI QuantStudio Sequence Detection System (Applied Biosystems, UK). Transcript levels of each gene were normalized to *ACTIN2* for Arabidopsis and *ACTIN11* for peanut. The relative expression levels were calculated using the 2^−△△Ct^ method^12^. All qRT-PCR analysis was performed using biological triplicate samples. Sequences for *Aradu.G22I6* (encoding ribulose bisphosphate carboxylase), *Aradu.53538* (encoding light-harvesting chlorophyll B-binding protein 3), *Aradu.ZV73M* (encoding the CHLH subunit of magnesium chelatase), *Aradu.Z9Z80* (encoding a glutamyl-tRNA reductase family protein), and *Aradu.LW197* (encoding chlorophyllide A oxygenase) were retrieved from PeanutBase (https://www.peanutbase.org/). Primers for qRT-PCR are listed in Table S2.

### Antibody preparation, protein extraction and immunoblotting

This experiment was conducted using previously described methods^56^. The *AhGLK1* coding sequence were cloned in the pGEX4T-1 vector to allow production of GST-AhGLK1 fusion proteins after induction by isopropyl β-D-1-thiogalactopyranoside (IPTG). Expression of GST-AhGLK1 was induced in *Escherichia coli* BL21-Codon Plus-RP (Agilent Technologies) by adding IPTG to a final concentration of 0.5 mM at 37 °C for 9 h, after which the bacteria were transferred to 16 °C overnight. GST-AhGLK1 protein was purified and used for antibody production (polyclonal, rabbit). The antibody of PORA was obtained from Agrisera (http://www.agrisera.com). Detection of AhGLK1 and AhPORA proteins by immunoblot analysis was carried out as previously described^57^. Leaves (100 mg) of peanut or Arabidopsis were ground in liquid nitrogen and homogenized with 1 mL of sample buffer (50 mM Tris, pH 6.8, 2 mM EDTA, 10% w/v glycerol, 2% SDS and 6% 2-mercaptoethanol), then denatured at 100 °C for 5 min, and finally subjected to SDS-PAGE.

### Cloning of *AhPORA* promoter and luciferase assay

Genomic DNA was isolated from peanut leaf samples as described^58^. The *AhPORA* promoter was obtained by PCR with the genomic DNA as template using specific primers (F: 5′-ccgctcgagGAGTTCCTTGTTGGGTTATGAC-3′, R: 5′-ggactagtGCAGAGCGATTGAAACCTTGG-3′). Reaction conditions were as follows: initial denaturation at 94 °C for 2 min, followed by 30 cycles of denaturation at 94 °C for 10 s, annealing at 55 °C for 30 s and extension at 72 °C for 1 min, with a final extension at 72 °C for 10 min. The PlantCARE database (http://bioinformatics.psb.ugent.be/webtools/plantcare/html/) was used to identify potential *cis*-acting elements within the promote^59^. The MegAlign. Version 7.1.0 of the DNASTAR software package (DNASTAR, Madison, WI) was used to analyze protein sequence alignments and the phylogenetic tree based on the Jotun-Hein alignment of amino acid sequences^60^.

To generate the firefly luciferase reporter construct, the *AhPORA* promoter was cloned into the pGreenII 0800-LUC vector. The *AhGLK1* cDNA was ligated into the pCanG vector, the effector vector in the luciferase assay. The Arabidopsis genotypes used in this assay were the WT ecotype. Arabidopsis leaves were incubated in 10 mL protoplast enzyme solution containing 1.5% cellulase, 0.75% macerozyme, 0.5 M mannitol, 10 mM MES pH 5.7, 10 mM CaCl_2_, 0.1% BSA on a shaking platform at 50 rpm for 3 h at 26 °C in the dark. The protoplasts were shown to be intact and viable by microscopy (Olympus BX51, Japan). Then 10 mL W5 solution (1.54 mM NaCl, 125 mM CaCl_2_, 5 mM KCl, 2 mM MES pH 5.7, 5 mM glucose) was added, followed by centrifugation at 60 rcf for 5 min at 4 °C. The protoplasts were washed three times, suspended in W5 solution, and placed on ice for 30 min. The supernatant was removed and MMG (15 mM MgCl_2_, 4 mM MES pH 5.7, 0.4 M mannitol) was added to form a protoplast suspension. Five µg reporter and 5 µg effector plasmids were co-transformed into 100 µL Arabidopsis mesophyll protoplasts with 110 µL PEG-CaCl_2_ solution (100 mM CaCl_2_, 0.2 M mannitol, 40% PEG 4000) with a 10 min incubation at room temperature. The transformed protoplasts were diluted in 220 µL W5 solution, followed by 440 µL and 880 µL W5 solution, after which protoplasts were harvested by centrifugation at 60 rcf for 5 min at 4 °C. The pellet was washed twice with W5 solution, resuspended in 100 µL W5 solution, and incubated for 16 h at room temperature in the dark. Dual luciferase assay was performed with the Dual-Luciferase Reporter Assay System kit (Promega, China) according to the instructions. The luciferase activity was measured using a microplate luminometer (Turner BioSystems, Sunnyvale, CA). The mean values (±SE) were calculated from three replicates.

### Statistical analysis

To evaluate the differences in data, the Student *t*-test was used. Quantitative data were expressed as mean ± SD. Means were compared using one-way analysis of variance or the Student *t*-test with SPSS19.0. Significance was assigned at *P* < 0.05.

## Electronic supplementary material

Supplementary Information
