# Supplementary material for: AhGLK1 affects chlorophyll biosynthesis and photosynthesis in peanut leaves during recovery from drought
Source: Sci Rep. 2018 Feb 2;8:2250. doi: 10.1038/s41598-018-20542-7 (PMC5796971; doi:10.1038/s41598-018-20542-7)
Supplement: Supplementary file 1 — Supplementary Information [file 41598_2018_20542_MOESM1_ESM.pdf]

AhGLK1 affects chlorophyll biosynthesis and photosynthesis in peanut leaves during recovery from drought

Xing Liu+, Limei Li+, Meijuan Li, Liangchen Su, Siman Lian, Baihong Zhang, Xiaoyun Li, Kui Ge, Ling Li*

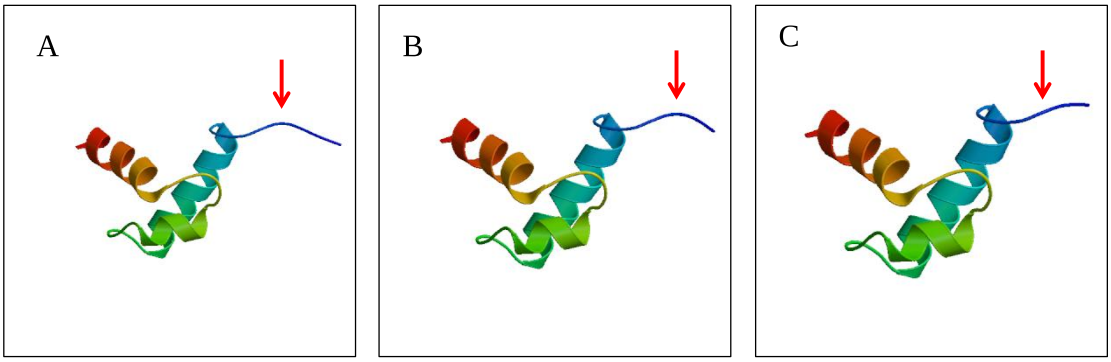

Figure S1. (A) AhGLK1, (B) AtGLK1 and (C) AtGLK2 structures as protein ribbon diagrams.
Differences are indicated by red arrows.

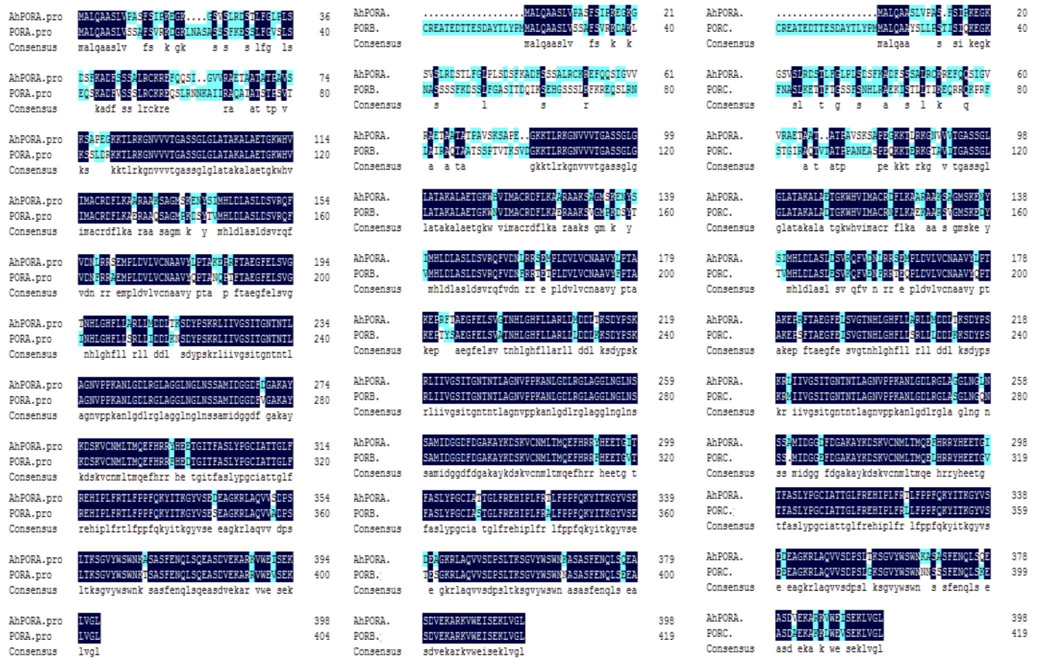

Figure S2. AhPORA protein sequence alignment with PORA (left), PORB (centre) and PORC (right) of Arabidopsis.

AhPORA exhibited 82.96%, 75.00%, and 72.68% identity with PORA, PORB, and PORC, respectively.

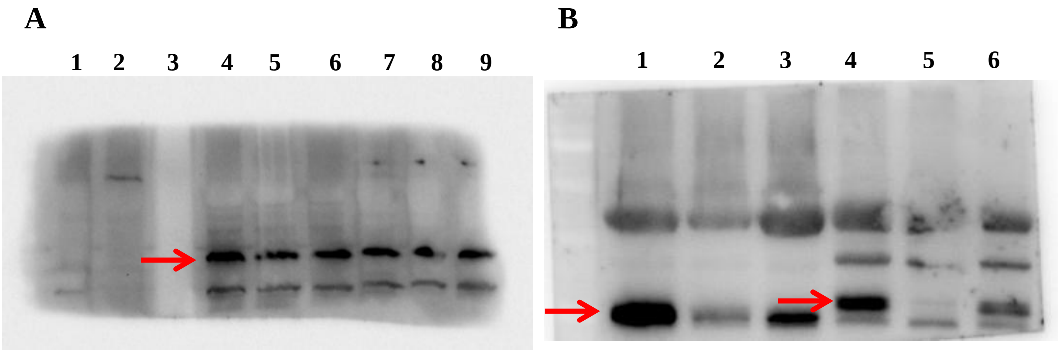

Figure S3. Western blots

A: AhGLK1 bands of peanut. 1-3: other samples; 4: Control; 5: Drought; 6:Recovery; 7-9: other samples. B: PORA bands of peanut (1-3) and Arabidopsis (4-6). 1: Control; 2: Drought; 3: Recovery; 4: WT; 5: *glk1glk2*; 6: *AhGLK1/glk1glk2*

Cropped blots are indicated by red arrows, the other blots were not special blots. Exposed for 10 S by GelDoc™ XR (Bio-rad).

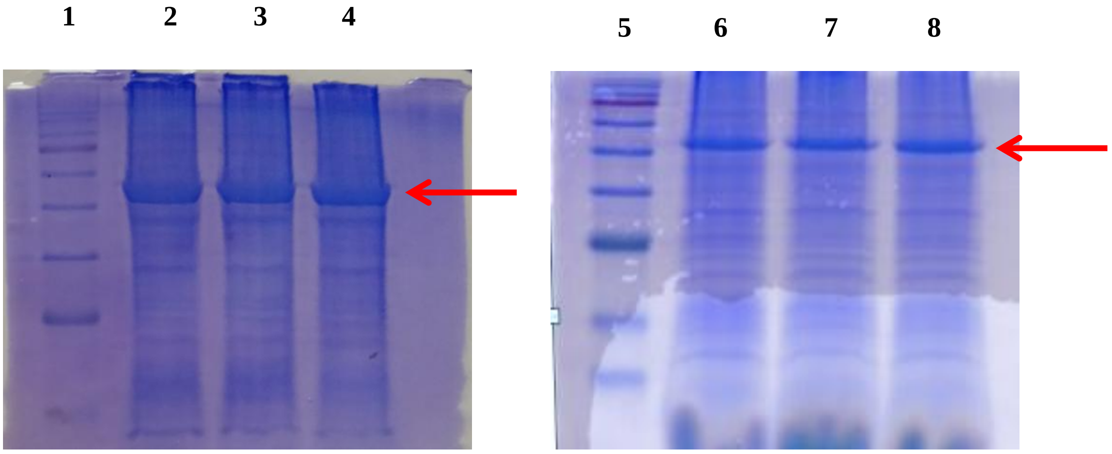

Figure S4. Loading control gel

1. Marker (10-180 kDa); 2. Control; 3. Drought; 4. Recovery; 5. Marker (10-180 kDa); 6. WT; 7. *glk1glk2*; 8. *AhGLK1/glk1glk2*

Cropped blots are indicated by red arrows.

Table. 1 Types and number of regulatory elements of *PORA* promoter in different species

Function	Site	Number of regulatory element				
		Arachis hypogaea	*Cajanus cajan*	*Cicer arietinum*	*Vigna angularis*	*Glycine max*
Light responsive element	G-box, MRE, Box I, GAG-motif, as-2-box, AAAC-motif, Box 4, TCT-motif, sp1, ACE	2	6	5	2	2
Cis element involved in abscisi cacid responsiveness	ABRE	1	1	1	1	1
Ethylene-responsive element	ERE	0	1	0	0	0
Auxin-responsive element	TGA-box	0	1	0	1	1
MeJA-responsive element	TGACG-motif	0	1	0	1	1
salicylic acid-responsive element	TCA-element	0	1	0	0	0
cis element involved in low temperature responsiveness	LTR	0	1	0	0	0
cis element involved in drought-inducibility	MBS	0	2	0	0	0
cis element involved in defense and stress responsiveness	TC-rich	0	0	0	1	0
cis element involved in anaerobic induction	ARE	0	0	1	0	0
cis element involved in heat stress responsiveness	HSE	0	0	1	1	0
protein binding site	Box III	0	0	0	1	1
MYBHv1 binding site	CCAAT-box	0	1	0	1	1
MYB Binding Site	MBS	0	0	0	1	0

Table 2. Primers used for qRT-PCR in this study

Primer name	Sequences (5'– 3')
ACTIN2-F	ggtaacattgtgctcagtggtgg
ACTIN2-R	aacgaccttaatcttcatgctgc
LHCB2.1- F	ggctgttcaagttgtgctca
LHCB2.1- R	gagaacatggcaagacgacc
LHCB3-F	ggttcaaagccggttcacaa
LHCB3-R	cgttgatgcggaaaccttca
LHCB6-F	aactataccggcgatcaggg
LHCB6-R	ttcagcctctccagcttctc
HEMA1-F	cagcacgggtttacaatgtg
HEMA1-R	gaatccctccatgcttcaaa
GUN4-F	atgcgttttaagccatcctg
GUN4-R	tgctcctactcctgcctgtt
CAO-F	gatctcgaccaccgagacat
CAO-R	gcgtcgtcttcagaatctcc
PORA-F	gggttgaacgggctaaacag
PORA-R	tatgccggtgtcttcatgga
ACTIN11-F	gggtgaggggaacattactg
ACTIN11-R	gagagacacactaccctttc
AhGLK1-F	tcgctagccaccttcagaaa
AhGLK1-R	ttccctctccctccaccata
Aradu.LW197 -F	ccttggttctgtaaatgagggtcgt
Aradu.LW197 -R	agcagtcgagtggatggcattttct
Aradu.Z9Z80 -F	gtggaatccctgtttcagaactttg
Aradu.Z9Z80 -R	ctcccagcacaagagagtcaagacc
Aradu.ZV73M -F	tctgaatgcggatggagagaacctg
Aradu.ZV73M -R	ctgcaaatccatgatgatgtgggctagc
Aradu.G22I6 -F	accgtaatatgcttggatg
Aradu.G22I6 -R	tgatgacttctggtggat
Aradu.53538-F	gagaagtgggtcagagtt
Aradu.53538-R	tttccaaggtagtcaagc
AhPORA-F	tcttctggactaggactggc
AhPORA-R	ttctcctagacatgccagc
